# How Delayed Cord Clamping Saves Newborn Lives

**DOI:** 10.3390/children12050585

**Published:** 2025-04-30

**Authors:** Judith Mercer, Elisabeth Saether, Tekoa King, Holger Maul, Holly Powell Kennedy, Debra Erickson-Owens, Ola Andersson, Heike Rabe

**Affiliations:** 1 College of Nursing, University of Rhode Island, Kingston, RI 02881, USA; debeo@uri.edu; 2 Møre and Romsdal Hospital Trust, 6026 Ålesund, Norway; elisabeth.sether@helse-mr.no; 3 School of Nursing, University of California, San Francisco, CA 94143, USA; tekoaking@gmail.com; 4 Department of Obstetrics and Gynecology of the Asklepios Kliniken Barmbek, Wandsbek and Nord-Heidberg, 22039 Hamburg, Germany; h.maul@asklepios.com; 5 School of Nursing, Yale University, West Haven, CT 06516, USA; holly.kennedy@yale.edu; 6 Department of Neonatology, Skåne University Hospital, 22185 Malmo/Lund, Sweden; ola.andersson@med.lu.se; 7 Department of Clinical Sciences, Pediatrics/Neonatology, Lund University, 22362 Lund, Sweden; 8 Brighton and Sussex Medical School, University of Sussex, Brighton BN1 9QG, UK; heike.rabe@nhs.net; 9 Department of Neonatology, University Hospitals Sussex NHS Foundation Trust, Royal Sussex County Hospital, Brighton BN2 5BE, UK

**Keywords:** blood volume, cord clamping, hypovolemia, ischemia, inflammation, jaundice, mechanotransduction, neonatal transition, placental transfusion, progesterone, sustained cord circulation

## Abstract

Interest in the subject of umbilical cord clamping is long-standing. New evidence reveals that placental transfusion, facilitated by delayed cord clamping (DCC), reduces death and need for blood transfusions for preterm infants without evidence of harm. Even a brief delay in clamping the cord shows improved survival and well-being, but waiting at least two minutes is even better. We propose that three major benefits from DCC contribute to reduced mortality of preterm infants: (1) benefits from the components of blood; (2) assistance from the continued circulation of blood; and (3) the essential mechanical interactions that result from the enhanced volume of blood. The enhanced blood volume generates mechanical forces within the microcirculation that support the newborn’s metabolic and cardiovascular stability and secure short- and long-term organ health. Several unique processes prime preterm and term newborns to receive the full placental transfusion, not to be misinterpreted as extra blood or over-transfusion. Disrupting cord circulation before the newborn’s lung capillary bed has been fully recruited and the lungs can replace the placenta as a respiratory, gas-exchanging organ may be harmful. Early cord clamping also denies the newborn a full quota of iron-rich red blood cells as well as valuable stem cells for regeneration, repair, and seeding of a strong immune system. We propose that delayed cord clamping and intact-cord stabilization have the potential to save lives by protecting many neonates from hypovolemia, inflammation, and ischemia.

## 1. Introduction

*“Another thing very injurious to the child, is the tying and cutting of the navel string too soon; which should always be left till the child has not only repeatedly breathed but till all pulsation in the cord ceases. As otherwise the child is much weaker than it ought to be, a portion of the blood being left in the placenta, which ought to have been in the child” Erasmus Darwin, 1801, [1] (p. 302).*

Interest in the subject of umbilical cord clamping is long-standing. Over 200 years ago, Charles Darwin’s grandfather shared his recommendation (above) based on his experiences, like those of many midwives and other practitioners who routinely delay cord clamping. As birth moved from home to the hospital, knowledge was lost about some “old-fashioned” practices such as early initiation of breastfeeding, early skin-to-skin contact between the mother and the newborn, and delayed cord clamping (DCC). More recently, evidence shows that these practices are safe and important for the health of newborns and mothers. The most recent meta-analyses demonstrate that a longer delay (>120 s) of cord clamping can save more preterm infants’ lives [2]. How and why does DCC work? This article describes the physiology of DCC and explains how sustained umbilical cord circulation after birth facilitates a large auto-transfusion from the placenta to the infant that potentially saves newborn lives.

The birth and neonatal transition processes are “gestalts” (organized wholes) that are much more than the sum of their parts. To understand how placental transfusion saves newborns’ lives, we will examine the larger picture—the interactions of the various facets of the neonatal transition at the level of the organism, tissue, and cells. This systems approach reflects the complexity of the transition process [3]. Reductionist science focuses on reducing complex situations into finer and finer parts. We have learned much from reductionism, but it has been unable to prevent or cure many difficult newborn problems such as respiratory distress syndrome, pulmonary hypertension, bronchopulmonary dysplasia, retinopathy of prematurity, and hypoxic–ischemic encephalopathy. In contrast, a systems approach examines how the pieces work together to support the whole system. This approach may help us to ask “different” research questions to identify how to best support newborn health and improve ongoing quality of life [4].

The transfer of placental blood via delayed cord clamping provides several advantages that likely decrease preterm mortality. We propose three major benefits from DCC that contribute to reduced mortality of preterm infants. These include (1) benefits from the components of blood; (2) assistance from the continued circulation of blood; and (3) the essential mechanical interactions that result from the enhanced volume of blood. Mechanical forces within the microcirculation support the newborn’s metabolic and cardiovascular stability and secure short- and long-term organ health. The fetal body is prepared for the enhanced blood volume by high progesterone levels [5,6]. After birth, high pulmonary artery pressures provide maximum perfusion throughout the newborn’s body [7]. These processes likely fill the newborn’s brainstem and other organs, prevent hypovolemia and subtle or overt ischemia, and help the newborn regain homeostasis after the stress of birth [8]. We provide evidence for each facet and explain how they work together to ensure newborn health, as well as examine the high cost of immediate (ICC) or early cord clamping (ECC).

## 2. Supporting Evidence: Reduction in Death After DCC for Preterm Infants

Multiple randomized controlled trials have reported that waiting to clamp the umbilical cord for at least 60 s after birth results in a 30% decrease in the incidence of death for preterm infants [9,10,11]. Yet, the most recent evidence about DCC shows that the current standard recommendations to delay clamping for 30 to 60 s may be insufficient and that longer delays result in fewer deaths [2].

A new systematic review and an individual participant data (IPD) meta-analysis of more than 6000 preterm infants have confirmed the finding of reduced mortality [2,12]. Authors of eligible studies were invited in 2017 to join the Cord Management at Preterm Birth Collaboration (iCOMP) and to share their IPD data [13]. New studies were added as they were completed. In the first of two publications (2023) from the iCOMP, the authors found that DCC, compared to ICC, reduced death before discharge with an odds ratio of 0.68 [95% CI 0.51–0.91]. This included 20 studies with 3260 infants and 232 infant deaths (high certainty) [12]. For secondary outcomes, they found less need for blood transfusion [DCC vs. ICC, 1136/2128 infants, OR 0.59] (high certainty) and a slight increase in hypothermia on admission to the NICU by −0.13 °C (−0.23 °F), OR 1.28, [CI 0.2–0.06] with moderate certainty.

In a companion paper, the authors compared the short, medium, and long delays of cord clamping with umbilical cord milking (UCM) and ICC in 6094 participants from 47 trials [12]. They found that a longer delay (>120 s) reduced death before discharge even more (OR 0.31, 95% CI 0.11–0.80, moderate certainty). These two iCOMP publications show that placental transfusion for preterm infants has significant benefits and minimal harm.

No studies exist among term infants to evaluate death after ICC, likely due to the low event rate. A recent international cluster-randomized crossover trial of UCM (milking the cord from the placental end to the newborn four times) versus ICC that included more than 1000 non-vigorous infants reported a significant reduction in hypoxic–ischemic encephalopathy (HIE) in the UCM group [14]. With four milkings, an infant would likely receive about 1/3 more of their placental blood volume compared to ICC [15,16]. This study is the first to suggest that allowing the non-vigorous infant more of its placental transfusion may prevent HIE. Although UCM does not provide for sustained cord circulation, it is likely a valuable prelude to longer DCC for infants older than 28 weeks’ gestation [17].

## 3. Physiology of Placental Transfusion at Birth

**Figure 1:**
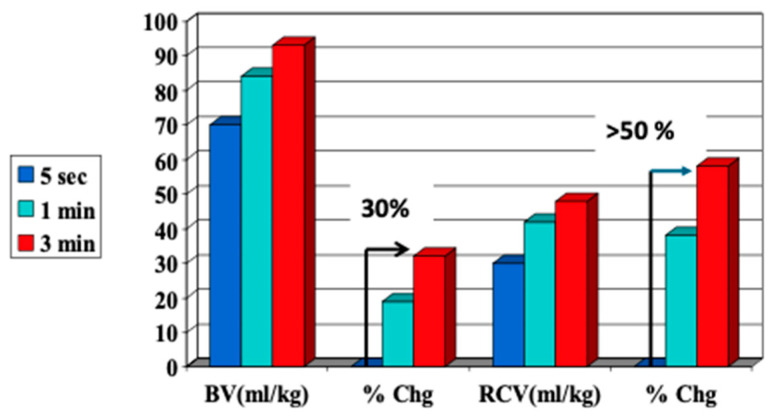
Percent change in blood volume and red cell volume caused by delayed cord clamping. Using 111 healthy term infants, held at the level of the perineum or below, Yao showed that a 3 min delay in cord clamping resulted in a 30% increase in blood volume and a 50% increase in RBCs. BV—blood volume; RCV—red cell volume. X axis—BV and RCV percent change or mL/kg. Y axis—percent. Arrow—percent change. Developed from data found in [18].

Placental transfusion occurs after birth and refers to the net transfer or redistribution of the infant’s own blood from the placenta to the newborn through the intact umbilical cord. Placental circulation, on the other hand, begins in early pregnancy and continues throughout. It can continue supporting the infant after birth for several minutes or more if the cord is left unclamped. While placental transfusion and placental circulation occur simultaneously after birth, albeit at differing rates, we discuss each process separately to aid in understanding (see sustained cord circulation in Section 5). Placental transfusion is usually facilitated by delaying cord clamping for several minutes after birth, with longer delays resulting in larger transfers (Figure 1) [2,18].

When the umbilical cord is left intact immediately after birth, blood circulates between the newborn and the placenta for several minutes. It will gradually transfer essential blood components into the newborn as the uterus contracts around the placenta [19]. An intact cord allows sustained cord circulation and physiologic and respiratory support [20]. These steps gradually enhance the newborn blood volume, providing mechanical interactions that facilitate essential mechanotransduction for the proliferation and growth of organs and tissues.

During pregnancy, the fetus’s blood circulates through its body and the placenta, driven by the fetal heart [21]. About 30% of the fetal blood is estimated to flow through the placenta at any point in time for term infants. With ICC, approximately 30% of the blood volume and 50% of the red blood cell (RBC) volume stay in the placenta (Figure 1) [18]. About 50% of early preterm newborns’ blood volume flows through the placenta due to the 1:1 placenta-to-body ratio. With ICC or ECC, the smallest preterm newborns can lose up to 50% of their blood volume [22,23]. Zhou et al. reported that even after 60 s of DCC for preterm newborns, they could drain 19 mL/kg of residual fetal/placental blood or about 25% of the blood volume potentially available for the preterm newborns.

The total fetal/placental circulation was estimated at 110–115 mL/kg by Linderkamp in 1982 [24]. There are currently no non-invasive ways to measure blood volume in the newborn infant, so this figure has not been validated recently. No provider knows at birth how much blood is in the placenta of any given newborn and how much is in its body. Infants who experience cord compression, for instance, could have much more than 30% sequestered in the placenta. When residual placental blood volume is measured after cord clamping, the amounts obtained vary greatly, even when the cord clamping time is the same [25]. Thus, the optimal time of cord clamping is not known, stressing the importance of sustained cord circulation to allow time for stabilization and equilibrium to be reached.

The practice of delaying cord clamping provides a gentle physiological transition as the infant adjusts to extrauterine life. Placental transfusion, in varying amounts, can be achieved by other methods, as seen in Table 1. However, the terms and nuances raise many questions [26]. Table 1 provides the varied terms and definitions to describe different methodologies related to placental transfusion that will be used in the following sections.

Throughout pregnancy, the placenta serves as an exteriorized organ system (respiratory, intestinal, endocrine, and immune) while the fetal organs develop. Most fetal organs do not produce, store, or secrete the specific substances that they will be responsible for after birth [27,28]. Thus, most fetal organs can develop and grow during pregnancy without the extra demand to be fully functional. At birth, the perfusion to each organ must expand rapidly to satisfy the increased demand for and consumption of oxygen and glucose. The blood in the placenta needs to move into the newborn’s lungs, intestines, and other organs that must now assume their genetically determined roles. The alveolar capillaries, not perfused in fetal life, require a considerable amount of blood to facilitate gas exchange and carbon dioxide (CO_2_) removal via the lungs [29]. Similarly, the gut, relatively silent during fetal life, now needs rapid perfusion of a large amount of blood as it assumes the functions of absorption and digestion [28]. Reducing blood volume and flow puts preterm infants at risk of lung and gut tissue injury secondary to hypoperfusion and hypoxia [28]. An overview of the functions of blood in the human body is found in Table 2.

Both the fetus and the mother have high levels of progesterone in the circulation [6]. This prepares the fetus’s body and organs to receive the large blood volume needed to fulfill the functions mainly performed by the placenta. All the blood in the fetal placental system belongs to the fetus, as there is no “extra” blood in this system. If the cord is clamped immediately (ICC), 30 to 50% of the fetal/placental blood volume can stay in the placenta and be lost to the newborn along with the rich components in the blood. ECC of 30 to 60 s can still significantly decrease the amount of blood to the newborn [23]. Thus, the amount of placental transfusion, with essential components of blood, that an infant can receive varies depending on cord management.

## 4. Placental/Cord Blood and Its Components

Placental/cord blood is at body temperature and is oxygenated [30,31]. It contains cell-rich plasma, fetal red blood cells (RBCs), stem cells, and other unique substances as listed in Table 3. The 20–40 mL/kg of plasma expands volume and includes many hormones (endocrine, pituitary- and hypothalamic-like), cytokines, proteins, messengers, immunoglobulins, and other endogenous components to support the healthy beginning of life as a newborn [32]. ICC can negatively impact these essential substances.

**Figure 2:**
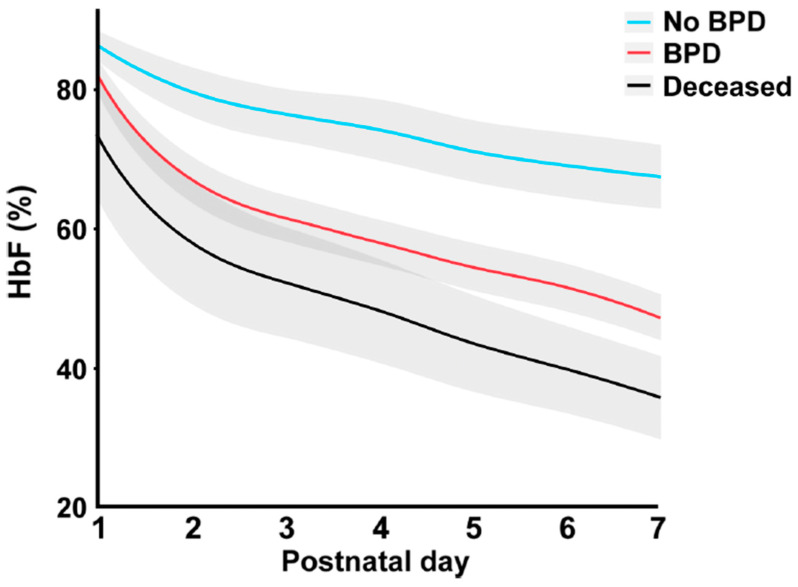
Mean levels (%) of HbF during postnatal days 1–7 in relation to the development of BPD and non-survival. A 10% increase in HbF was associated with a decreased prevalence of BPD, OR 0.64 (95% CI 0.49 to 0.83; *p* < 0.001). Infants with no BPD (n = 169) in light blue; infants with BPD (n = 213) in red; infants who died prior to PMA 36 weeks (n = 61) in black. The shadowed area illustrates a 95% CI. During the first weeks of life, much of the endogenous blood with important fetal components is exchanged for adult blood due to clinical intervention. Minimizing early postnatal loss of endogenous blood components and maintaining levels of HbF may be essential for the prevention of BPD in the very preterm. BPD, bronchopulmonary dysplasia; HbF, fetal hemoglobin; PMA, postmenstrual age. From Hellström W, et al. Open Access: Creative Commons CC BY 4.0. [34].

The RBCs in placental/cord blood contain fetal hemoglobin (HbF), which is the only molecule that carries oxygen (O_2_) throughout the body and transports CO_2_ for excretion. More RBCs result in increased oxygen-carrying capacity. Fetal RBCs have two important unique properties compared to adult RBCs: more flexible membranes, which allow for a better fit in the fetus/newborn’s tiny capillaries, and HbF, which helps to prevent oxidative damage by not releasing oxygen as quickly [33]. This may be why higher levels of HbF in infants’ blood are associated with better health and survival, while low levels are associated with health conditions such as bronchopulmonary dysplasia, retinopathy of prematurity, and death (Figure 2) [34]. The 50% increase in RBC volume provided by a full placental transfusion contains enough iron to meet an infant’s needs for several months since RBCs contain 70–80% of the iron in our bodies [35,36].

Placental/cord blood contains many naïve (undifferentiated or blank) stem cells that seed the newborn’s body. These constitute a built-in repair system. Preterm infants have the highest percentage of stem cells circulating in their blood [37]. Stem cells replenish cells throughout the body and last a lifetime. At birth, the stem cells found in placental/cord blood provide “nature’s first transplant”, providing a unique opportunity to seed tissues throughout the body to build immune, blood-making, and other systems [38,39]. Providing sustained cord circulation allows time for the gradual transfer of these important components for newborn and lifetime health.

## 5. An Intact Cord Offers Continued (Sustained) Blood Circulation

*“Is it possible that this wonderful alteration in the human machine [neonatal transition]...could be properly brought about in one instant of time, and at the will of a by-stander?” Charles White, 1791, [40] (p. 109)*

When the umbilical cord is left intact after birth, sustained cord circulation (SCC) can provide a backup or redundancy support during the first several minutes of life [20]. SCC facilitates a continuous exchange between maternal and newborn circulation and allows the transfer of the special components in cord blood (Section 4), as well as the volume required to induce mechanical signals within the vascular system (Section 6). SCC provides placental gas exchange to support the newborn while gradually adapting to air breathing and recovering from the stress of labor and birth [41,42,43]. It allows time to perfuse all organs with oxygenated blood, including the brainstem, which is the seat of all normal reflexes, especially breathing and consciousness [44]. In addition, it assists with the removal and dilution of acidic lung fluid from the newborn’s lungs into the general circulation [45,46]. SCC facilitates clearance of fetal/newborn metabolic byproducts by the placenta and removal of excessive stress-related metabolites accumulated in labor [47]. Continued communication with the placenta likely creates a low-pressure reservoir that prevents overshoots of blood pressure [48]. Sustained cord circulation may also provide thermal support, as the cord blood holds body temperature when leaving the placenta [30,31]. The newborn remains a fetus until the cord is clamped, ending placental circulation, at which time it must begin to survive on its own.

Sustained cord circulation also allows time for intact cord stabilization (ICS) and supports an infant during resuscitation (ICR). Supporting evidence from both experimental (animal) and observational (human) studies indicates that the cord should not be clamped before ventilation is established, as this results in a smoother cardio-vascular transition and may improve survival [49,50]. While there is much focus on establishing ventilation, the other functions mentioned above, which may take more time to accomplish, are also essential for long-term health. This contrasts with those who believe that once the newborn has breathed, there is no usefulness in continuing SCC. Traditionally, midwives have valued the resuscitative capabilities of the placenta, postponing cord clamping until cessation of cord pulsations or after the cord collapsed, or even after placental delivery [51]. In recent years, this approach has again been actualized by the introduction of mobile, bedside resuscitation equipment [52].

A special variant of SCC and intact-cord stabilization implies delivering the placenta before/without cord clamping during caesarean section, also defined as extra-uterine placental transfusion/perfusion (EPT). The method was described 75 years ago as a life-saving intervention for preterm infants delivered by caesarean section [53]. It has later been tested for feasibility and safety in both term and preterm caesarean deliveries [54,55,56,57]. Although this method keeps the umbilical cord intact until ventilation is established, it may be argued that the placental gas exchange is disrupted after removal of the placenta. However, a recent randomized controlled trial on very preterm infants found better cerebral and peripheral oxygenation at five minutes applying EPT compared to DCC (≥30 s) [58]. A plausible explanation may be that EPT yields better organ perfusion due to a longer period from delivery to cord clamping, and that sustained cord circulation may be equally important during stabilization as placenta-to-infant gas exchange [59].

Supporting evidence shows us that resuscitation of the non-vigorous newborn at birth with the cord intact (ICR) results in improved outcomes for infants [60]. This placental/cord blood does NOT constitute “extra” blood for the newborn but is its own blood used throughout fetal life and needed for ongoing growth and development. Sustained cord circulation provides time for the transfer of enhanced blood volume needed to provide mechanical forces.

## 6. Mechanical Interactions Resulting from Enhanced Blood Volume

*“It is not widely appreciated that the dysfunction of the inner lining of blood vessels is the single most common cause of human mortality. Endothelial cells (ECs) control their micro-environments as gatekeepers of organ development, homeostasis, and tissue regeneration”. Hellmut Augustin, 2017, [61] (p. 1).*

Mechanotransduction is the biological mechanism by which cells sense, integrate, and respond to physical forces in their environment [62]. It is a crucial aspect of physiological function necessary for survival in all living organisms, from single-celled bacteria to complex animals. It converts a mechanical force, such as pressure, strain, or shear force, into a biochemical signal that causes a biological response [63,64,65]. Essentially, every cell needs to be able to sense and interpret mechanical stimuli to maintain its structure and function properly. This process influences most biological functions.

**Figure 3:**
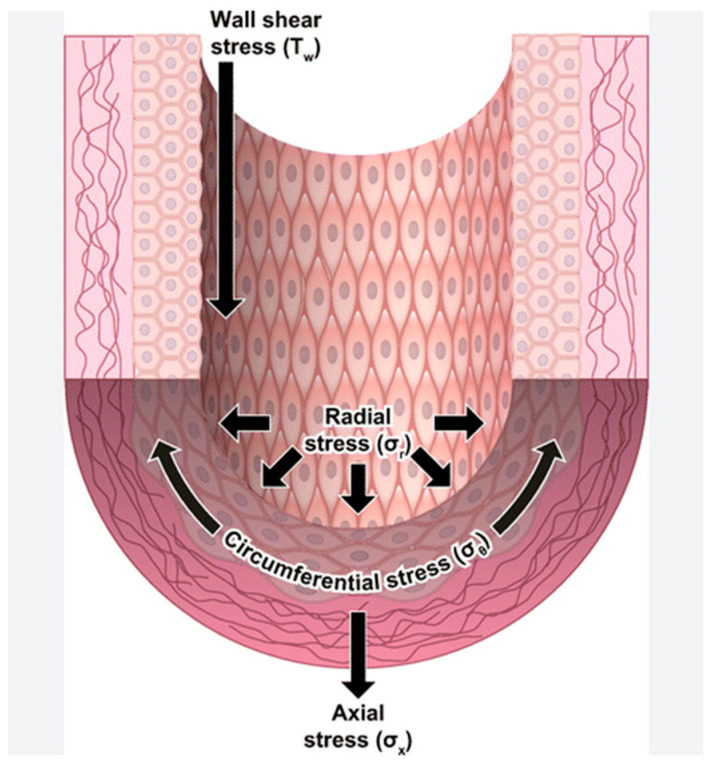
Example of mechanical forces (wall, radial, and circumferential stress) from blood flow acting on the arterial wall. From Davis MJ et al. (Figure 1, p. 1248). Used with permission from [66].

One essential mechanical force comes from constant and dynamic blood flow, resulting in a pulsatile stretch that provides shear force in all directions (circumference, radial, and wall shear stress) (Figure 3) [66].

This internal force on blood vessel walls results in vital intracellular actions. Hemodynamic forces significantly impact the cardiovascular system, beginning with sculpting the developing fetal heart and blood vessels [67]. Mechanical forces also contribute to the regulation of vascular tone and vascular remodeling, as well as local blood flow [62,64].

Capillaries, lined by ECs and extensively embedded in all organs, are vital in assuring our survival and well-being. Capillaries are not just passive conduits for blood circulation. The mechanical force from enhanced blood flow and pulsatile stretch causes capillary ECs to secrete tissue-specific growth factors that affect many physiological processes [68]. These growth factors, especially the angiocrine factors produced by ECs, maintain organ homeostasis, balance the self-renewal and differentiation of stem cells, direct postnatal tissue remodeling, and oversee organ regeneration [69,70].

**Figure 4:**
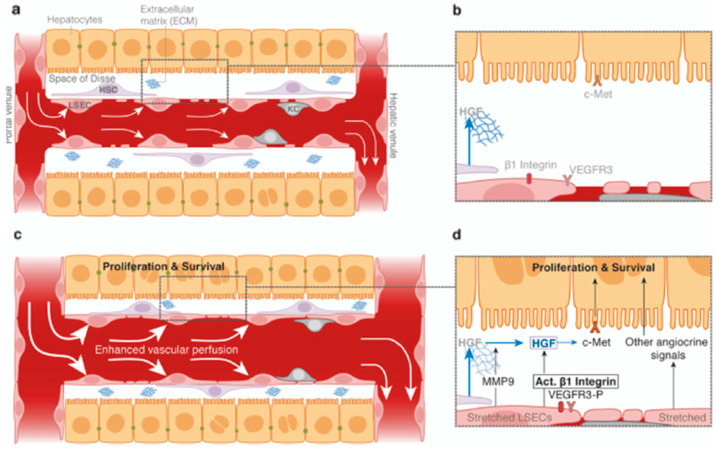
Model of mechanotransduced angiocrine signals in the liver. Shown are simplified drawings of liver sinusoids (specialized capillaries) with their specific ECs and hepatocytes (liver cells). (**a**) shows liver sinusoid under normal to low blood flow, while (**c**) illustrates a liver sinusoid with enhanced blood perfusion flowing through it. (**b**) shows an enlarged part of the space of Disse, a narrow space between the capillary and the liver cell, where plasma can penetrate, and action takes place between blood and tissue. A magnification of the space of Disse is represented in (**b**,**d**). In (**b**), the ECs (at the bottom) are not stretched, and there is no action. In (**d**), the ECs are circumferentially stretched by the widening of the vascular lumen and thus activated to release angiocrine and paracrine factors. These factors enhance the proliferation and survival of the adjacent hepatocytes (liver cells). Similar processes are present in many other organs. Used with permission from [68].

In a landmark study by Lorenz (2018), the researchers demonstrated that mechanical forces alone stimulate the process of secreting angiocrine factors [68]. Figure 4 is a simplified drawing showing liver sinusoidal cells and epithelial cells at work with limited perfusion (a) compared to enhanced perfusion (c). The ECs are stretched by enhanced perfusion, producing angiocrine factors that stimulate activity in the Space of Disse (d), enhancing the proliferation and survival of adjacent cells and, in turn, the organ itself. Further details can be found in Appendix A.

Understanding one organ system can inform us about others. Morrisey notes that “many of the intercellular signaling networks controlling the morphogenesis of the respiratory system are conserved and used in the development of other branched tubular organs…” [71] (p. 2). The mechanotransduction process is not unique to liver cells; it occurs throughout all body parts [70]. Likely, the enhanced blood volume from a full placental transfusion and the resultant enhanced perfusion may provide a vital mechanical force necessary for normal growth and development in all the infant’s rapidly growing organs and tissues. Normal growth and development are significant, as an infant’s most rapid period of growth occurs in the first few months after birth, as the birth weight doubles.

### Clinical Example of Enhanced Perfusion from a Full Placental Transfusion

Pietra and Oh (1968) provided an informative clinical example of the effect of cord clamping times on heel perfusion [72]. They performed heel biopsies on 12 newborns, 6 of whom had long DCC (3–5 min delay held at or below the perineum) and 6 who experienced ICC. In the infants with ICC, histologic slides showed more undilated capillaries with thick endothelia, very small lumens, and no fenestrations. In contrast, the slides from the heels of infants who had DCC showed more fully dilated capillaries with wide open lumens and thin endothelia in the heels of the newborns. These dilated capillaries had several fenestrations, allowing for excellent exchange of gases and nutrients. One can assume that if the heels were better perfused in the newborns with longer DCC, the lung, gut, brain, and other organs would also have had improved perfusion, providing a protective effect from subtle or overt inflammation and ischemia following hypovolemia.

Mechanotransduction within the microcirculation is a key factor in how DCC reduces the death of preterm infants. The enhanced blood volume generates mechanical forces within the microcirculation that support the newborn’s metabolic and cardiovascular stability and secure short- and long-term organ health. Redistribution of the newborn’s blood volume from the placenta to all organs that must now grow, and function independently provides the essential mechanical interactions. DCC likely prevents morbidity and/or death for some preterm newborns and improves the quality of life for others. Other unique processes that support this critical period of neonatal transition are discussed below.

## 7. Other Supporting Processes

Several unique processes prepare preterm and term newborns to receive an enhanced blood volume from placental transfusion. High progesterone levels, complex lung changes, and high pulmonary artery pressure enable the neonate’s body to receive and incorporate the large blood volume into the previously minimally functioning organs and tissues.

### 7.1. Vascular Relaxation from High Levels of Progesterone

Progesterone, a potent vasodilator produced mainly by the placenta, plays an essential role in preparing the infant to accommodate the large volume of blood circulating in the placenta just before birth [6]. High levels of circulating progesterone assist in essential vascular remodeling and relaxation of vessels during the newborn transition to accommodate the incorporation of essential blood volume [73]. It is also primarily responsible for the 50% increase in maternal blood volume during pregnancy. At birth, progesterone levels in the newborn (~270 ng/mL) are even higher than in the mother (~170 ng/mL) [6]. The high level supports the essential rapid transition within the newborn circulatory system by assuring the vascular and tissue plasticity of blood vessels necessary for the prompt expansion and new perfusion of body tissues and organs. Progesterone is also highly neuroprotective for the fetus and newborn and is known to diminish responses to inflammatory cytokines [5].

At no other time, short of severe hemorrhage, could one transfuse such a large amount of blood into a human body. This blood volume level cannot be infused later since the rapid loss of progesterone over the first 12 h of life reduces vessel plasticity. The quick fall of the elevated progesterone levels over the first 12 h of life demonstrates the conserved timing of its unique influence. This specific pattern of progesterone levels supports the theory that birth and the first day of a newborn’s life constitute a critical period in organ development.

### 7.2. Lung Expansion by Air Versus Capillary Distension with Blood

After birth, the lungs must rapidly change from a fluid-filled organ to an organ of gas exchange. This process begins at birth with recursive increases in blood perfusion to the alveolar capillaries, creating capillary erection [74]. Capillary erection provides a scaffolding for the distension of alveoli, allowing air to enter the alveoli more easily and protecting the fragile lung tissue, and may be the mechanism that is essential to prevent damage to the delicate lung tissue [75,76]. The stretching of the alveolar capillaries due to increased perfusion causes Type 2 alveolar cells to release surfactant, reducing surface tension in the alveoli [77].

A prevailing belief has been that the newborn lung must be opened with high initial positive pressure to decrease the pulmonary vascular resistance and force lung fluid out of the alveolar spaces [78]. However, recent evidence shows inflammation and injury to the delicate lung tissue by attempts at ventilation before perfusion of the alveolar capillaries. Tingay states that “even the most protective ventilation strategy applied to preterm newborns in the delivery room causes inflammation and injury to the preterm lung” [75]. Katheria (2016) and Meyer (2021) have shown that most preterm newborns breathe spontaneously in the first minute of life without assistance [79,80]. Newborn response (startling) suggests forced ventilation is painful [81,82]. We suggest a gentler approach of allowing sustained cord circulation that will provide oxygenated blood needed to stimulate the brainstem respiratory centers to initiate spontaneous breathing, essential to open the newborn’s airway [83]. These findings and the available knowledge that the placenta continues to function as a respiratory organ in the first minutes after birth suggest that the belief that the lung must be aerated rapidly at birth is incorrect and possibly damaging [76].

### 7.3. High Pulmonary Artery Pressures Are Found with Good Perfusion

The high pulmonary artery pressure (PAP) reported in newborns with a full placental transfusion helps to perfuse all of the newborn’s organs copiously [7]. Along with progesterone, it facilitates the essential stretch needed for post-birth organ transition. The increased blood volume delivers significant mechanical force to induce essential transformation in the newborn’s vascular system at the level of capillary endothelia [67,68]. It supports the assimilation of the large blood volume, formerly traversing the placenta, into the newborn’s newly functioning organ systems. In human infants living at sea level, pulmonary artery pressure falls to adult levels within two weeks, the significant decline occurring in the first 2 to 3 days after birth [84]. The increased blood volume from the placenta transfers to the newborn’s organs as they begin to function while adapting to extrauterine life.

Paradoxically, later high PAP is associated with lung pathology, where narrowed, damaged alveolar blood vessels make it harder for the heart to pump blood through them, raising pressure [32]. ICC contributes to the endothelial damage of the alveolar capillaries due to the lack of blood volume to dilate them fully [85].

## 8. Concerns About Potential Adverse Effects of DCC

The most frequent concerns providers have voiced about placental transfusion are the risk of jaundice and the possibility of over-transfusion, although the recent literature supports neither. The risk of jaundice, hyperbilirubinemia, and the need for phototherapy from “extra” RBCs following DCC appears to be exaggerated since the idea rests on conclusions from a meta-analysis heavily weighted by an unpublished study performed more than 30 years ago [86]. No increase in hyperbilirubinemia requiring treatment has been reported in recent randomized trials, even when the subjects were newborns with alloimmunization [87,88,89]. Elevated serum bilirubin is a physiological response after birth. Bilirubin is a potent antioxidant, and higher (but within normal) levels may offer neuroprotection to infants [90].

Although a hematocrit greater than 65% does occur in some infants following DCC, symptomatic polycythemia has not been reported even in growth-restricted newborns [36,91]. It is possible that during studies of DCC versus ICC, trial results could be biased if pediatric providers are not blinded to the randomization of participating infants [92].

Some providers have voiced concern that newborns may receive too much blood from a full placental transfusion. However, the fetus has been using this entire fetal/placental blood volume throughout pregnancy to grow and develop normally. It needs the blood volume to switch from its placental “temporary” organs to its “lifelong” organs. A full placental transfusion cannot “over-transfuse” the newborn, as the fetal heart has been circulating all this blood throughout its body and back and forth to the placenta to meet the fetus’s metabolic needs. Loss of ~30% of its blood volume immediately after birth results in the equivalent of a moderate to severe hemorrhage, which could contribute to the higher death rate with ICC [93]. Understanding how the newborn is primed in utero for this transfusion should ease concerns about over-transfusion.

## 9. The High Cost of Immediate or Early Cord Clamping

*“Endothelial dysfunction is an important factor in the pathogenesis of acute and chronic diseases of prematurity including…essentially all morbidities that occur in premature infants” Dwayne Mascarenhas, 2024, [94] (p.3).*

ICC or ECC reduces blood volume in the newborn. It contributes to the potential loss of organ-specific vascular competence in the gut, brain, kidney, and other organs, resulting in morbidity and mortality. ICC and/or ECC were developed for efficiency and expediency without scientific evidence [51,95]. They remain a common practice in the US and worldwide despite the current recommendations for a delay of “at least” 30 to 60 s for cord clamping [96,97,98]. The most recent research suggests that these recommendations are insufficient or are based on a limited understanding of the underlying physiological and pathophysiological processes [12]. Interestingly, a longer delay should be indicated for severe obstetrical complications, such as shoulder dystocia, breech delivery, or a tight cord around the neck, often accompanied by hypovolemia developing at birth [99,100]. Compression of the umbilical cord leads to a reduction of the venous, O_2_-enriched blood flow from the placenta to the fetus, resulting in placental pooling and fetal hypovolemia. These babies are often compromised at birth, appearing pale, atonic, and areflexic. Some can be asystolic. ICC allows the limp, hypovolemic infant to be passed quickly to the neonatologist, even though those newborns might benefit most from DCC via intact cord resuscitation (ICR) [101,102,103]. Experts need to re-examine the ICC policy for depressed infants. Several recent studies report improved outcomes for infants with ICR [42,104,105].

### Hypovolemia, Inflammation, and Ischemia

Deficiency in fetal/placental blood volume following ICC or ECC can result in a 30–50% loss of effective circulatory volume, potentially putting preterm infants at high risk for hypovolemic shock, inflammation, ischemia, and death. Hypovolemia leads to inadequate perfusion of tissues, creating an imbalance between oxygen demand and the body’s ability to supply it. When there is decreased intravascular volume, the circulation may not match the tissues’ demand for oxygen, creating a potentially life-threatening condition [106]. The body compensates for volume loss by increasing the heart rate and contractility, followed by baroreceptor activation, which results in costly sympathetic nervous system activation with vasoconstriction.

Reduced blood volume stimulates inflammation, resulting in hypoxic–ischemic damage [107,108,109,110]. When ECs are damaged, a systemic inflammatory response directly affects the brain, lungs, liver, GI tract, and even bone marrow [111]. Ischemia can follow, resulting in loss of organ-specific vascular competence, which likely leads to many common newborn diseases [28,34,94,112].

Depletion of the normal circulating blood volume not only results in decreased provision of glucose to the tissues but also in ischemia. There is a rapid decrease in short-lived, highly reactive molecules that store and produce energy, followed by a loss of hemostasis from the impairment of the many membrane-bound ionic pumps [113]. A high rise in intracellular calcium leads to activation of deleterious enzyme systems and intracellular signaling pathways [114]. Reduced tissue pH occurs secondary to lactate accumulation. Hypoperfusion resulting in severe hypotension can lead to intracranial hypertension as it impairs normal regulatory processes of the cerebral circulation [115]. Cardiac output declines due to decreased ventricular filling, and systolic blood pressure drops. Because of sympathetic nervous system activation, blood flow is redirected to the heart and brain and away from noncritical organs and tissues. While this protects heart and brain function, it leads to further oxygen deprivation in other tissues, causing more lactic acid production and worsening acidosis [106]. The worsening acidosis, along with hypoxemia, leads to ischemia and eventually causes the loss of peripheral vasoconstriction, worsening hemodynamic compromise, and potentially death [116]. We suggest that an enhanced blood volume from sustained cord circulation via intact cord stabilization and/or resuscitation could protect many neonates from these physiological insults.

## 10. Summary

DCC can provide major benefits for infants that help to reduce mortality and morbidity. Leaving the cord intact allows sustained cord circulation that disperses the many essential, rich components of newborn blood while facilitating enhanced blood volume. The enhanced blood volume provides mechanical forces that stimulate capillary endothelial cells to release angiocrine growth factors that support the newborn’s metabolic stability and initiate proper organ health. Even a brief delay in clamping the cord shows improved survival and well-being, with longer delays offering more benefit. A full blood volume, provided by DCC, is likely essential for normal growth, health, and tissue regeneration throughout the newborn’s body and for the reduction of morbidity and mortality.

## Figures and Tables

**Table 1:** Defining terms related to placental transfusion.

Term	Definition
Placental Transfusion	Redistribution of blood between the placenta and the newborn results in a net auto-transfusion if cord circulation is sustained (not clamped). Amounts of blood vary depending on cord management. General term—needs a clear definition in any study or guideline.
Optimal Cord Management (OCM)	Waiting until the cord is flat and white and/or the placenta is ready to deliver. Often misused without defining exactly how the cord was managed.
Immediate cord clamping (ICC)	Clamping the cord as soon as possible or “immediately” after birth, often before breathing has commenced. In some papers, it is used to mean before 30 s.
Early cord clamping (ECC)	Clamping before 30 s up to <one minute. Often used for control groups in studies of cord management.
Delayed Cord Clamping (DCC)	Delaying (or deferring) the clamping of the cord for various times ranging from “at least 30 to 60 s” or 10 min or more. Always needs to be defined for each use.
Umbilical Cord Milking (UCM)	Manually milking (squeezing) the umbilical cord from the perineum to the newborn, releasing and repeating up to four times. UCM can be performed with the cord intact (iUCM) or it can be cut as long as possible before milking (cUCM).
Physiological-Based Cord Clamping (PBCC)	Clamping after regular breathing is established or when the preterm infant is stabilized (HR > 100, SpO_2_ > 85%, using <40% supplemental oxygen).
Intact Cord Stabilization (ICS)	Allowing the infant to stabilize while the cord is still attached to the placenta, as long as needed.
Intact Cord Resuscitation (ICR)	Leaving the cord intact for the non-vigorous infant while resuscitation is ongoing, and often after stabilization.
Sustained cord circulation (SCC)	Allows for bi-directional blood flow, where an individual equilibrium can be reached for an optimal transition to occur.
Extrauterine placental transfusion (EPT)	Removing the placenta along with the newborn at cesarean section without cord clamping to allow for further placental transfusion to take place exteriorly of the uterus.
Residual Placental Blood Volume	Used to approximate the volume of placental transfusion by manually draining the residual blood remaining in the placenta after delivery and reporting the volume in milliliters.

**Table 2:** Functions of blood.

Rationale	Function
(1) Transfer of blood components	Transport of different blood cells
	Transport of stem cells
	Transport of hormones and enzymes
	Protection against pathogens
	Protection against blood loss (hemostasis)
(2) Continued circulation of blood	Transport of respiratory gases (oxygen, carbon dioxide)
	Transport of nutrients and metabolic wastes
	Regulation of pH and electrolytes
	Regulation of body temperature
(3) Mechanical interactions due to enhanced blood volume	Regulation of fluid (blood) volume and mechanotransduction
	Regulation of organ and tissue perfusion and proliferation

**Table 3:** Essential placental/fetal blood components and their functions.

Components	Functions
**Plasma**	
Water	Serves as a transport medium; carries heat
Electrolytes	Major role in membrane excitability; buffers pH changes; aids osmosis between blood and extracellular fluid
Nutrients (glucose, proteins), wastes, gases, and hormones	All are transported in blood; blood CO_2_ influences acid–base balance
Plasma proteins Synthesized by the liver	Provides an osmotic effect important for the distribution of fluids between vascular and interstitial compartments
Albumin	Contributes to colloid osmotic pressure; a transporter
Globulins	Crucial for immune defense and various bodily processes
Alpha and beta globulin	Function as enzymes; transport many water-soluble substances, proteins, clotting factors, and precursors
Gamma globulin	Helps the immune system fight infections and foreign substances
Fibrinogen	Supplies fibrin meshwork for a clot
**Cellular Components of Blood**	
Red blood cells (erythrocytes)	Transports O_2_ to cells and CO_2_ to the placenta to be excreted
White blood cells	Part of the immune system—fights infections and diseases
Neutrophils	Engulfs bacteria and debris; 1st line protection
Eosinophils	Role in allergic reactions
Basophils	Release histamine and heparin
Monocytes	In transit to become macrophages
Lymphocytes	Produce plasma proteins
B lymphocytes	Produce antibodies
T lymphocytes	Produce a cell-mediated immune response
Platelets	Contribute to hemostasis; necessary for coagulation
Stem cells	Foundation for all cells, tissues, and organs in the body—maintain and renew tissues (regeneration)

## Data Availability

Not applicable.

## Abbreviations

The following abbreviations are used in this manuscript:

CO_2_—carbon dioxide; DCC—delayed cord clamping; ECC—early cord clamping; ECs—endothelial cells; HbF—hemoglobin F (fetal); ICC—immediate cord clamping; iCOMP—Cord Management at Preterm Birth Collaboration; ICR—intact cord resuscitation; RBC—red blood cell.

## Appendix A

Figure 4 Lorenz et al. study details: Using murine fetuses, Lorenz noted that perfusion to the liver increased greatly just before a major growth period. Liver expansion was preceded by initial new blood perfusion in a highly controlled manner. They found higher levels of activated forms of two important angiocrine growth factors, β1 integrin and vascular endothelial growth factor receptor 3, in the periphery of the liver where there was extensive blood perfusion and hyperproliferation of hepatocytes. They found that activation of ECs could be decreased or increased by lowering or increasing the fetal heart rate pharmacologically. To test activation of ECs in human cells, the group mechanically stretched human liver cells. They found that stretching alone caused activation of the ECs. Next, they cultured new human liver cells in a medium derived from the stretched liver cells and found that the new cells experienced increased proliferation and decreased apoptotic death. The factors seen within **d** include β1 integrin that regulates cell behavior and interacts with VEGFR3 (vascular endothelial growth factor receptor 3), a protein that helps establish and maintain the body’s lymphatic and cardiovascular systems. C-Met (specialized protein) binds to hepatic growth factor (HGF), which is responsible for growth and repair. MMP9 (matrix metalloproteinase) remodels the intracellular matrix to allow for growth.
